# Antibiotic Resistant *Salmonella* and *Vibrio* Associated with Farmed *Litopenaeus vannamei*

**DOI:** 10.1100/2012/130136

**Published:** 2012-04-24

**Authors:** Sanjoy Banerjee, Mei Chen Ooi, Mohamed Shariff, Helena Khatoon

**Affiliations:** ^1^Institute of Bioscience, Universiti Putra Malaysia, Selangor, 43400 Serdang, Malaysia; ^2^Faculty of Veterinary Medicine, Universiti Putra Malaysia, Selangor, 43400 Serdang, Malaysia

## Abstract

*Salmonella* and *Vibrio* species were isolated and identified from *Litopenaeus vannamei* cultured in shrimp farms. Shrimp samples showed occurrence of 3.3% of *Salmonella* and 48.3% of *Vibrio*. The isolates were also screened for antibiotic resistance to oxolinic acid, sulphonamides, tetracycline, sulfamethoxazole/trimethoprim, norfloxacin, ampicillin, doxycycline hydrochloride, erythromycin, chloramphenicol, and nitrofurantoin. *Salmonella enterica* serovar Corvallis isolated from shrimp showed individual and multiple antibiotic resistance patterns. Five *Vibrio* species having individual and multiple antibiotic resistance were also identified. They were *Vibrio cholerae* (18.3%), *V. mimicus* (16.7%), *V. parahaemolyticus* (10%), *V. vulnificus* (6.7%), and *V. alginolyticus* (1.7%). Farm owners should be concerned about the presence of these pathogenic bacteria which also contributes to human health risk and should adopt best management practices for responsible aquaculture to ensure the quality of shrimp.

## 1. Introduction

Shrimp is an important commodity in the global fishery trade due to its increasing demand and competitive international price [1]. As a result of rising shrimp exports, traditional shrimp farming which began in Malaysia in the 1930s has given way to intensive farming system. At present, the bulk of the production consists of *Litopenaeus vannamei* having a production of 52,926 tonnes [2]. It has been introduced in the early 2000 due to the advantages in terms of disease management and most widely cultured in intensive system throughout Malaysia for local consumption as well as for export. 

With the change to intensive culture system having high stocking density, disease problems appear frequently causing heavy economic losses to the industry. Antibiotics are normally used to prevent or treat disease outbreaks in shrimp farming [3]. However, extensive use of antibiotics in shrimp farming can cause the development of antibiotic-resistant pathogens which can infect both cultured animals as well as humans [4, 5]. 

Shrimp intended for export have to meet the bacteriological standards of the importing countries. *Salmonella *and *Vibrio* species are important foodborne pathogens and most importing countries do not accept them in raw frozen shrimp. Contamination of tropical shrimp with *Salmonella* due to growth in polluted waters has been a problem in many parts of the world and is reported to be a part of the natural population of the brackishwater cultured shrimp [6]. In the United States, most *Salmonella* contamination problems in seafood were in shrimp [7]. In addition, opportunistic *Vibrio* spp. is the most common bacterial pathogen found in shrimp which can cause lethal infections following primary infections with other pathogens, environmental stress, nutritional imbalance, and/or predisposing lesions [8]. In humans, *Vibrio* spp. are known to cause gastroenteritis, cholera, and septicemia [9].

Therefore, the present study was carried out in major shrimp producing areas to identify the incidence of *Salmonella *and *Vibrio *in *Litopenaeus vannamei* cultured in commercial shrimp farms and their resistance to some of the commonly used antibiotics in aquaculture.

## 2. Materials and Methods

### 2.1. Sampling

Clinically healthy with no external lesions or clinical signs and alive *L. vannamei * were collected from three farms from growout ponds (80–120 days; total 6 ponds) situated in Carey Island (2°52′0′′ N, 101°22′0′′ E) and Kuala Selangor (3°21′0′′ N, 101°15′0′′ E), Malaysia. The live shrimp were transported in oxygenated pond water filled plastic bags and put into another plastic bag filled with ice flakes and placed in styrofoam boxes. Water samples from ponds were collected in sterile bottles and transported in ice. Samples were transported to the Aquatic Animal Health Unit in Universiti Putra Malaysia and processed immediately for examination. The three farms had well laid out ponds and had characteristics as shown in Table 1.

### 2.2. Bacterial Isolation

#### 2.2.1. Shrimp Sample

For isolation of *Salmonella*, the head and exoskeleton of shrimp were removed aseptically. Muscle and intestine (10 g) were separately taken aseptically from the shrimp, placed in individual sterile test tubes, and homogenized in 3 mL autoclaved seawater using stomacher for 1 m. The homogenized sample was incubated in buffered peptone water at 37°C for 24 h for preenrichment. Selective enrichment was done using 10 mL of Rappaport and Vassiliadis (RVS) broth (Merck, Germany) inoculated with 1 mL culture from buffered peptone water and incubated at 42°C for 24–48 h. A loopful of sample from RVS was then streaked on selective media xylose-lysine-tergitol 4 (XLT-4) (Merck, Germany) and brilliant-green phenol-red lactose sucrose (BPLS) agar (Merck, Germany) and incubated at 37°C for 24–48 h. Subculture on XLT-4 and BPLS was done to obtain pure culture. *Salmonella* spp. colonies appear black or black-centered on XLT-4 and red centered pink on BPLS. 

For isolation of* Vibrio* from shrimp, one loopful of haemolymph was taken from hepatopancreas and cultured in thiosulphate citrate bile salt sucrose (TCBS) agar (Merck, Germany) with 3% sodium chloride (NaCl) (Merck, Germany). The culture was incubated at 25°C for 18 to 24 h. Yellow and green single colonies were subcultured on TCBS and tryptic soy agar (TSA) (Merck, Germany) with 3% NaCl at 25°C for 24 h to obtain pure culture.

#### 2.2.2. Water Sample

Pond water (10 mL) was diluted with 90 mL buffered peptone water and processed according to shrimp samples for isolation of *Salmonella*. In the case of *Vibrios*, pond water was plated on TCBS agar using the spread plate technique and processed according to shrimp samples.

### 2.3. Bacterial Identification

Different biochemical tests such as triple sugar iron (TSI), urease test, lysine iron agar (LIA), sulfide indole motility (SIM), slide agglutination test, and *Salmonella* serotyping were performed for identification of *Salmonella* spp. 

*Vibrio* species identification was done using API 20E (bioMérieux, France) identification system. *Escherichia coli* (ATCC 25922) was used as control. Gram staining was also done from single pure culture colony.

### 2.4. Antibiotic Sensitivity Test

Antibiotic susceptibility test was conducted using disc diffusion method on Mueller Hinton agar at 37°C for 24 h. Procedure was based on the standardized disc agar diffusion method of the National Committee for Clinical Laboratory Standards for antimicrobial susceptibility tests [10]. After incubation, the diameter of the zone of inhibition was measured and compared with BBL zone interpretative chart to determine the sensitivity of the isolates to the antibiotics. The BBL zone interpretative chart was used in the absence of standard interpretative scheme for environmental isolates or for shrimp pathogens. The antimicrobials oxolinic acid 2 *μ*g (OA 2), compound sulphonamides 300 *μ*g (S3 300), tetracycline 30 *μ*g (TE 30), sulfamethoxazole 23.75 *μ*g/trimethoprim 1.25 *μ*g (SXT 25), norfloxacin 10 *μ*g (NOR 10), ampicillin 10 *μ*g (AMP 10), doxycycline hydrochloride 30 *μ*g (DO 30), and erythromycin 15 *μ*g (E 15) were selected as they are veterinary important antimicrobials. Chloramphenicol 30 *μ*g (C 30) and nitrofurantoin 300 *μ*g (F 300) were also included since they were in use few years ago. The antimicrobials were from Oxoid, UK.

## 3. Results

### 3.1. Bacterial Isolation and Identification

#### 3.1.1. Shrimp and Water Samples

One *Salmonella* and five *Vibrio* spp. were isolated and identified from shrimp and water samples. *Salmonella enterica *serovar Corvallis was isolated from water sample in Farm 1 and shrimp samples from Farm 2 and 3. However, five *Vibrio* spp., namely, *V*. *alginolyticus*, *V*. *cholera*, *V*. *mimicus*, *V*. *parahaemolyticus*, and *V*. *vulnificus* were isolated from both shrimp and water samples from all the three farms (Table 2). Farm 1 and 3 had low occurrence of *Vibrio* spp., while Farm 2 had moderate to high occurrence. The overall occurrence of *Salmonella* and *Vibrio* in shrimp was 3.33% and 48.3%, respectively.

### 3.2. Antibiotic Sensitivity Test

The *Salmonella enterica *serovar Corvallis isolates from water in Farm 1 and shrimp in Farm 3 were found to be resistant to erythromycin only. However, the two *S. enterica *serovar Corvallis isolates from shrimp in Farm 2 were found to be resistant to all antibiotics except for nitrofurantoin and norfloxacin (Table 3). 

In Farm 1, *V*. *vulnificus* and *V*. *mimicus* (S1, S2) isolated from shrimp were resistant to AMP 10. On the other hand, *V*. *mimicus* (S1, S2) isolated from water was resistant to SXT 25, S 300, and AMP 10, whereas *V*. *cholerae* (S1, S2) was resistant to AMP 10 only. 

In case of Farm 2, *V*. *cholerae* (S1, S3) isolated from shrimp were resistant to DO 30, AMP 10, and TE 30, *V*. *cholerae *(S2) to DO 30 and TE 30, whereas *V*. *cholerae *(S4, S5, S6) were resistant to AMP 10. *V*. *cholerae* isolated from water was resistant to AMP 10 only. Besides,* V*. *mimicus* isolated from shrimp and water, and *V*. *vulnificus* isolated from shrimp were found to be resistant to AMP 10 also. In Farm 3, all the vibrios isolated from shrimp and water were resistant to AMP 10 (Table 3). 

All *Vibrio* isolates except for two were resistant to ampicillin. Tetracycline was the second highest and doxycycline was the third highest that the *Vibrio* spp. were resistant to (Table 3). Farm 1 and 2 were found to have more antibiotic-resistant patterns (one to four antibiotics) than Farm 3 (one to two antibiotics). Out of the five *Vibrio* species, *V. cholerae* showed the most antibiotic-resistant pattern.

## 4. Discussion

Results of this survey showed that *Salmonella* and *Vibrio* isolated and identified from the three shrimp farms are a serious cause for concern since they are of public health significance. *Salmonella* is facultative anaerobes and belongs to the family Enterobacteriaceae, and more than 2500 serovars of *Salmonella* are considered potential pathogens in animal and human. Many *Vibrio *spp. are pathogenic to humans and have been implicated in foodborne disease. 

Several studies have been done on prevalence of *Salmonella* in the tropics [11–13]. In the present study, *Salmonella* was found in water samples from Farm 1 and shrimp samples from Farm 2 and 3. This is in accordance with the studies where *Salmonella* have been reported from shrimp pond water [1, 12, 14] and shrimp [11, 12, 15]. Studies by Iyer and Varma [16], Bhaskar et al. [11], and Wan Norhana et al. [12] emphasized that *Salmonella* is natural part of the microflora of the shrimp culture practice. However, the absence of *Salmonella* from water and shrimp samples in some farms leading to a low occurrence in the present study could mean that *Salmonella* is not a common normal flora in shrimp culture environment. This is in accordance with a study done in Thailand by Dalsgaard et al. [17] who reported the absence of *Salmonella* from shrimp, sediment, water, and pelleted feed. Study by Koonse et al. [14] also showed that *Salmonella* is not part of the natural flora of the shrimp culture environment or naturally present in shrimp growout ponds. It is related to the concentration of fecal bacteria in the source of water supply to the growout pond water. In the present survey, the water source for two of the shrimp farms were from Langat River. The Langat River is one of the principal rivers draining a densely populated and developed area of Selangor. The major pollution sources affecting Langat River are sewage treatment plants, manufacturing industries not equipped with proper effluent treatment facilities, livestock, and pig farms [18]. Therefore, farmers should treat the water properly before introducing into the culture ponds.

In the present study, all *S. enterica *serovar Corvallis isolated from shrimp and water showed resistance to erythromycin. This is in agreement with the study done by Wan Norhana et al. [12] where *S. enterica *serovar Weltevreden*, S. enterica *serovar Hvittingfoss*, S. enterica *serovar Litchfield*, S. enterica *serovar Agona*, S. enterica *serovar Paratyphi*, S. enterica *serovar Benin, and* S. enterica *serovar Java isolated from shrimp were resistant to erythromycin. However, *Salmonella* isolated from Farm 2 showed multiple antibiotic resistances (eight antibiotics) compared to Farm 1 and 3 which were resistant to one antibiotic only. The occurrence of multiple antibiotic resistances could be due to the presence of chicken farm nearby that maybe using different types of antibiotics. Antibiotic is used in poultry as therapeutic as well as growth promotant. According to Singer and Hofacre [19], antibiotics and their metabolites as well as bacteria can spread from poultry farms into waterways. In addition, poultry litter can also help in their dissemination onto open field. Petersen et al. [20] have reported that integrated broiler chicken-fish farm contributed to antimicrobial-resistant bacteria in a pond environment. The antibiotic residues from the nearby chicken farm could have led to multiple antibiotic resistance observed in the present survey. 

The natural occurrence of vibrios in marine and estuarine environment has been reported by Varnam and Evans [21]. Incidence of vibrios in marine-caught seafoods including shrimp has been reported by Adeleye et al. [22], while Boinapally and Jiang [23] showed that vibrios are also found in pond-reared shrimp. 

In general, the incidence of bacteria resistance to shrimp samples was higher than those in water samples from the same location. In the present survey, differences in antibiotic resistance patterns in a species of *Vibrio *could be due to presence of different strains. Bacteria resistance to AMP10 was the highest followed by TE30. Ampicillin is not commonly used in shrimp culture. So there is a possibility that these vibrios could have acquired resistance from other places. The widespread use of tetracycline because of its low toxicity and broad-spectrum antibiotic activity against a wide range of Gram-positive and Gram-negative bacteria [24] and also as a successful prophylaxis and therapy against *Vibrio* [25, 26] could have led to high resistance. 

In the present survey, although the managers of the farms stated that they did not use any antibiotics, the possibility of the presence of antibiotic-resistant bacteria in shrimp could be from postlarvae. Yasuda and Kitao [27] reported that *Vibrio *spp. were the dominant genera in the digestive tract of the zoea of *Penaeus japonicus*. According to Baticados and Paclibare [28], a variety of drugs are used in shrimp hatcheries. The use of these drugs leads to resistance to certain antimicrobials during the rearing of postlarvae in the hatchery which remain in the shrimp gut when transferred to the growout ponds [29]. 

The other possibility of the presence of antibiotic resistant bacteria could be the use of probiotics. According to Mathur and Singh [30], there are reports that commensal bacteria including lactic acid bacteria may act as reservoirs of antibiotic resistant genes that can be transferred to pathogenic bacteria. Therefore, the use of probiotics in the surveyed farms may have led to the incidence of multidrug resistant bacteria. 

Four out of five *Vibrio* spp. isolated in the present study were similar to the findings of Bhaskar and Setty [31] who reported the presence of *V. alginolyticus* as the most common followed by *V. cholerae, V. parahemolyticus*, and *V. vulnificus* in *P*.* monodon* culture system. Farm 3 had two *Vibrio* species more than Farm 1 and 2 that had three species each. This could be because of the different source of postlarvae or different source of water. Farm 1 and 2 obtained postlarvae from the same hatchery and had the same source of water. 

The result of this survey reveals the presence of multidrug resistant *Salmonella* and *Vibrio* in shrimp farms. Antibiotic resistance is a legitimate concern which may affect future therapy of shrimp and human disease. Farm owners should be concerned about the presence of these pathogenic bacteria which also contributes to human health risk and should adopt best management practices for responsible aquaculture to ensure the quality of shrimp.

## Figures and Tables

**Table 1:** Characteristics of the three farms from where shrimp and water were sampled.

	Farm 1	Farm 2	Farm 3
Average pond size (ha)	0.4	0.4	0.4
Stocking density (PL/m^2^)	80	80	80–100
Age of farm (years)	13	16	10
Number of ponds	20	14	52
Source of water	Brackish water river	Brackish water river	Brackish water sea
Use of antibiotics	No	No	No
Use of probiotics	No	Yes	Yes
Source of feed	Direct-feed miller	Direct-feed miller	Direct-feed miller
Nearby farms	No	Chicken farm	Shrimp farm
Market	Local	Local, export	Local, export

**Table 2:** Percentage (%) of *Salmonella* and *Vibrio* spp. in three farms isolated from shrimp and water.

Isolated bacteria	Farm 1		Farm 2		Farm 3	
	Shrimp (*n* = 60)	Water	Shrimp (*n* = 60)	Water	Shrimp (*n* = 60)	Water
*Salmonella enterica * Serovar Corvallis	0	Yes	10		3.3	
*Vibrio mimicus*	15	Yes	40	Yes	10	
*Vibrio vulnificus*	5		20		3.3	Yes
*Vibrio cholera*	0	Yes	60	Yes	16.67	Yes
*Vibrio parahaemolyticus*	0		0		20	Yes
*Vibrio alginolyticus*	0		0		3.3	

Isolates from water did not state percentage as only one sample was taken per pond to determine if the bacteria isolated came from water.

**Table 3:** Susceptibility (zone of inhibition in mm) of *Salmonella* and *Vibrio* species isolated from different shrimp farms to antibiotics.

	Isolates	NOR 10	DO 30	E 15	SXT 25	F 300	S3 300	AMP 10	OA 2	C 30	TE 30
Farm 1											

Shrimp	*V. mimicus *(S1)	S-31	S-24	MS-18	S-26	S-25	S-20	R-6.5	S-29.5	S-31	S-23.5
	*V. mimicus *(S2)	S-31	S-23	MS-19	S-24.5	S-23	S-30	R-6	S-28	S-32	S-22
	*V. mimicus *(S3)	S-28	S-17	MS-20	S-22	S-22	S-28	MS-16	S-22.5	S-29	MS-18
	*V. vulnificus*	S-18	S-21	MS-18.5	S-23.5	S-23	S-30	R-7	S-28	S-30	S-24
Water	*Salmonella*	S-40	S-21	R-12	S-18	S-24	S-30	S-24	S-26	S-26	MS-17
	*V. cholerae *(S1)	S-31	S-18.5	MS-20	S-24	S-23	S-24	R-6	S-30	S-31	S-20
	*V. cholerae *(S2)	S-26	S-22	MS-16	S-25	S-27.5	S-26	R-6	S-29	S-32	S-23
	*V. mimicus*	S-29.5	S-17	MS-20.5	R-6	S-23	R-6	R-7.5	S-25.5	S-29.5	R-13

Farm 2											

Shrimp	*Salmonella *(S1)	S-26	R-6	R-6	R-6	S-20	R-6	R-6	R-6	R-6	R-6
	*Salmonella *(S2)	S-28.5	R-8	R-6	R-6	S-21	R-6	R-6	R-6	R-6	R-6
	*V. cholerae *(S1)	S-29	R-9	MS-18	S-24	S-23.5	S-28	R-6	S-27	S-30	R-14
	*V. cholerae *(S2)	S-29	R-10	MS-16	S-23.5	S-21	S-22	MS-13.5	S-28.5	S-28	R-11.5
	*V. cholerae *(S3)	S-32	R-10.5	MS-18	S-27	S-24	S-25	R-6	S-30.5	S-31.5	R-10.5
	*V. cholerae *(S4)	S-29	S-20	MS-19.5	S-22.5	S-22	MS-15	R-7.5	S-27.5	S-31	S-19
	*V. cholerae *(S5)	S-26.5	MS-14	MS-17.5	S-22.5	S-21.5	S-27	R-7	S-24.5	S-32	MS-16
	*V. cholerae *(S6)	S-24	S-18	MS-16.5	S-20.5	S-20	S-27	R-6	S-24	S-29	MS-16
	*V. mimicus *(S1)	S-29	S-23	MS-18	S-24	S-24	S-22	R-6	S-29.5	S-35	S-25
	*V. mimicus *(S2)	S-28	S-22	MS-20	S-22	S-24.5	S-28	R-6	S-27	S-32	S-24
	*V. mimicus *(S3)	S-27.5	S-18.5	MS-20	S-25	S-24	S-31	R-13	S-29	S-31	S-21.5
	*V. mimicus *(S4)	S-26	S-21	MS-18.5	S-23	S-23.5	S-30	R-6	S-25	S-30	S-22
	*V. vulnificus *(S1)	S-28	MS-14	MS-19	S-24	S-22	S-28	R-8	S-25	S-30	MS-18
	*V. vulnificus *(S2)	S-26	S-20	MS-18.5	S-23	S-21.5	S-26	R-7.5	S-29	S-31	S-23
Water	*V. cholerae*	S-30	S-21	MS-20	S-26	S-23	S-22	R-8	S-28.5	S-30	S-19.5
	*V. mimicus*	S-27	S-16.5	MS-19.5	S-22.5	S-22.5	MS-14	R-6	S-24	S-29.5	S-20.5

Farm 3											

Shrimp	*Salmonella *(S1)	S-42	MS-15	R-10	S-25	S-23	S-20	S-26	S-28	S-25	S-20
	*Salmonella *(S2)	S-41	MS-13	R-10	S-27	S-21.5	S-20	S-26	S-27.5	S-25	MS-17
	*V. alginolyticus*	S-23	S-24	MS-17	S-20.5	S-23	S-17	R-6	S-22	S-32	S-24
	*V. cholerae *(S1)	S-25	S-24	MS-20	S-24	S-25	S-26	R-6	S-22	S-31	S-25
	*V. cholerae *(S2)	S-24	S-22.5	MS-19	S-22.5	S-24	S-25	R-6	S-19	S-30.5	S-25
	*V. cholerae *(S3)	S-21	MS-13	MS-16	S-22	S-22	S-27	R-7.5	S-27	S-26.5	R-14
	*V. cholerae *(S4)	S-24	S-19.5	MS-17.5	S-24	S-21	S-25	R-6	S-27.5	S-27	S-20.5
	*V. cholerae *(S5)	S-25	S-18	MS-15.5	S-23.5	S-19	S-24	R-6	S-27	S-26.5	S-21
	*V. mimicus *(S1)	S-27	S-22	MS-17	S-25	S-21.5	S-21	R-9	S-26.5	S-30	S-23
	*V. mimicus *(S2)	S-26	S-20	MS-17.5	MS-15	S-22	S-19.5	R-8	S-26.5	S-25	S-20
	*V. mimicus *(S3)	S-25	S-20	MS-16	S-18	S-18	S-25	R-10	S-24	S-28	S-21.5
Water	*V. parahaemolyticus *(S1)	S-28	S-23.5	MS-19	S-25	S-22	S-23.5	R-6	S-28	S-31	S-24.5
	*V. parahaemolyticus *(S2)	S-23	S-20.5	MS-17	S-21.5	S-20	S-25	R-8	S-25	S-30	S-21
	*V. parahaemolyticus *(S3)	S-24	S-22	MS-17	S-24	S-21	S-20	R-7.5	S-25	S-29	S-21.5
	*V. parahaemolyticus *(S4)	S-24	S-20.5	MS-16.5	S-21	S-20	S-19	R-9	S-24.5	S-30	S-20
	*V. parahaemolyticus *(S5)	S-22.5	S-18	MS-16.5	S-23	S-20	S-26	R-8.5	S-22	S-26.5	S-20
	*V. parahaemolyticus *(S6)	S-23	S-17.5	MS-18	S-20.5	S-19.5	S-25	R-12.5	S-23	S-28.5	S-20
	*V. vulnificus *(S1)	S-22.5	S-21	MS-17.5	S-22	S-24	S-22.5	R-6	S-22.5	S-28	S-21.5
	*V. vulnificus *(S2)	S-26	S-18.5	MS-16.5	S-16	S-22	S-26.5	R-9	S-27	S-28	S-20
	*V. cholerae*	S-30	S-26	S-24	S-27.5	S-30	S-28	R-7	S-25.5	S-33	S-28
	*V. parahaemolyticus *(S1)	S-29	S-23	MS-19.5	S-25	S-24	S-23.5	R-6	S-28	S-30	S-24
	*V. parahaemolyticus *(S2)	S-26	S-20	S-27.5	S-22.5	S-21	S-17	R-9	S-25	S-30	S-20.5
	*V. vulnificus*	S-34	S-30	S-24	S-36	S-32	S-40	R-10	S-30	S-40	S-34

NOR 10: norfloxacin 10 *μ*g; DO 30: doxycycline hydrochloride 30 *μ*g; E 15: erythromycin 15 *μ*g; SXT 25: sulfamethoxazole 23.75 *μ*g/trimethoprim 1.25 *μ*g; F 300: nitrofurantoin 300 *μ*g; S3 300: compound sulphonamides 300 *μ*g; AMP 10: ampicillin 10 *μ*g; OA 2: oxolinic acid 2 *μ*g; C 30: chloramphenicol 30 *μ*g; TE 30: tetracycline 30 *μ*g; S: susceptible; MS: moderately susceptible; R: resistant.
